# The transition to an activity-based workplace: Experiences of managers and employees from a sense of coherence perspective in public sector workplaces

**DOI:** 10.1371/journal.pone.0320324

**Published:** 2025-03-31

**Authors:** Josefine Hansson, Stig Vinberg, Erika Wall, Pär Löfstrand

**Affiliations:** 1 Department of Health Sciences, Faculty of Human Sciences, Mid Sweden University, Östersund, Sweden; 2 Department of Psychology and Social Work, Faculty of Human Sciences, Mid Sweden University, Östersund, Sweden; University of Gothenburg: Goteborgs Universitet, SWEDEN

## Abstract

**Background:**

The Activity-Based Workplace (ABW) is an increasingly popular office design that aims to facilitate new ways of working. Research focusing on the effects of ABWs on various outcomes is growing but there is a lack of studies looking at how managers and employees perceive the transition to ABWs from a salutogenic health-promotion perspective. This study aims to explore how managers and employees experienced a transition period to ABWs through the lens of a sense of coherence.

**Methods:**

A qualitative design was applied through semi-structured interviews with six managers and focus group interviews with nine employees working in the public sector in Sweden. A content analysis of the interviews was conducted using Aaron Antonovsky’s sense of coherence concept, with the three main aspects of comprehensibility, meaningfulness and manageability acting as a theoretical framework for the analysis.

**Results:**

Six sub-categories within the three main SOC categories were identified in the material and considered important to the participants during their transition to the ABW. The sub-categories were divided into facilitating factors and barriers to obtaining a sense of coherence. The identified factors were: information and preparation, clear rules*,* adaptability, leadership, social relations and health and well-being.

**Conclusions:**

The analysis showed that the managers and employees were exposed to factors which may have acted as both facilitators and barriers to a sense of coherence during the transition to the ABW. When implementing ABWs, consideration must be given to the facilitators and barriers identified in this study during the transition process. The sense of coherence framework appears valuable for gaining a deeper knowledge of managers’ and employees’ experiences during the transition to ABW.

## Introduction

The Activity-Based Workplace (ABW) is becoming increasingly popular. It is an office design that aims to facilitate new ways of working [1,2]. ABWs are often implemented in organisations due to cost savings, more efficient use of office space and increased flexibility [1]. The ABW recognises that throughout the day, workers engage in many different activities, and to accommodate these activities they can choose various types of work settings. No fixed workstations are assigned for the employees and instead, they are expected to transit between different work areas depending on the task they will perform [3,4]. For instance, a quiet area with few distractions can be chosen if a task requires concentration. If the work task requires closeness to colleagues, an open area for communication and collaboration may be selected [3].

Research focusing on the effects of ABWs on various outcomes is growing, although the findings are still inconsistent. For instance, positive work conditions have been found in ABWs, such as increased work satisfaction due to autonomy [5], better task collaboration across teams [6], increased physical activity and less back pain [7]. Conversely, negative impacts such as a higher level of distraction and cognitive stress in ABWs compared to cellular offices [8], musculoskeletal problems [2], lack of privacy [5], lack of communication within teams [9], decreased work engagement and fatigue [10] have been found. Mache et al. [11] found that perceived occupational stress decreased and perceived workload increased after the transition to ABWs from traditional offices in a large technology company [11]. A longitudinal study found that relocating to ABWs from traditional offices in a large Swedish government agency significantly decreased employees’ perceived productivity. However, the productivity loss among employees was reduced if leadership was perceived to be change-oriented. That study highlighted the importance of leadership behaviour and actions when implementing organisational changes [1]. Although research demonstrates both positive and negative consequences of ABWs for organisations, employees, and managers, these consequences vary depending on the nature of the work [12].

The majority of research on ABWs has focused on the employees’ experiences [3,9,13] rather than on leaders [1,14], and only a limited number of studies have integrated perspectives from both groups [15]. In addition, studies on the transition and implementation processes for both groups are rare [1,11].

Research on the combination of remote work and office-based work, including models such as ABWs in public sector workplaces, remains limited, as noted by Carrasco-Garrido and De-Pablos-Heredero [16]. This gap in knowledge forms the empirical foundation for the present study. The proportion of respondents who partially or fully work from home varies significantly across European countries, ranging from approximately 20% to 80%. Sweden stands out as one of the countries with the highest prevalence of people working from home, with around 60% of workers engaging in this type of work [17], which has contributed to a greater adoption of alternative office solutions.

Public sector workplaces constitute the largest segment of the workforce in many countries. In Sweden, approximately 1.5 million individuals are employed in the public sector, out of a total workforce of around 5 million [18]. Within the Nordic countries, the adoption of new public management strategies and the reduction of available resources have been posited as contributing factors to the increased workload and reduced job control experienced in public sector environments [19]. In Swedish public sector workplaces, the psychosocial working conditions are frequently challenging, resulting in significant health issues and elevated rates of sick leave among employees [20–22]. In addition, extensive research on managers within the Swedish public sector [21,23,24] highlights that many managers face arduous working conditions, often exacerbated by a lack of adequate organisational support structures.

The concept of sense of coherence (SOC) can offer valuable insights for a better understanding of how people navigate the transition to ABWs. The SOC framework was developed by Antonovsky [25] and reflects the coping capacity of people to handle everyday life stressors. A strong SOC makes a person cognitively and emotionally capable of understanding problems and willing to face them. On the other hand, a weak SOC decreases a person’s ability to cope with difficult situations effectively. The SOC encompasses three components – comprehensibility, meaningfulness and manageability. Comprehensibility is a measure of the ability to perceive incidents and circumstances as structured and understandable. Meaningfulness is the belief that challenges and demands are worthy of investment. Manageability is the ability to cope with difficult situations and have sufficient internal and external resources to do so [25]. The term *generalised resistance resources* (GRR) is closely connected to the SOC and refers to the resources of a person, a group or a community that enable the individual’s abilities to cope effectively with stressors and contribute to the development of an SOC [26]. These may be resources such as knowledge, coping strategies, social support, the individual’s state of mind and material resources. It was initially believed that a person’s SOC stabilised in early adulthood, but it was later proposed that it can be further influenced by life experiences, such as the working environment [25]. Workers with a strong SOC feel confident that resources are available to cope with work demands and are more likely to adopt adaptive strategies that are suitable to the specific situation [27,28]. If adequate GRRs are present, challenges are resolved, leading to a strengthened SOC. On the other hand, if GRRs are lacking or inappropriate, challenges remain unresolved or are resolved poorly leading to a weaker SOC [29].

A large number of studies indicate that a strong SOC is associated with better health and well-being in different people and groups and that it is cross-culturally applicable [30–32]. A strong SOC is also positively related to other health resources, such as resilience, optimism and coping [30]. In the context of work, a high SOC has been associated with better self-reported health and greater work engagement among nurses [33], and an enhanced ability for managers to mobilise and generate social resources in the workplace, leading to perceptions of a supportive organisational climate [34]. Longitudinal analyses have also found that the SOC might protect against the negative effects of organisational change on mental health [35]. Furthermore, in a Finnish study, a good organisational climate and low job insecurity were related to a strong SOC, which in turn was associated with a high level of well-being in employees working in different organisations. The aforementioned study also concluded that various environmental changes may cause changes in the SOC.

In the context of ABWs, research focusing on the SOC is scarce, and to our knowledge, only one study has examined the SOC of people working in ABWs. The longitudinal study by Wijk et al. [36] found that a higher SOC was associated with better health, well-being and satisfaction during relocation to ABWs in the public sector in Sweden. In addition, the negative effects of relocation on work satisfaction were mitigated by higher levels of perceived meaningfulness. The authors recommended conducting additional studies on ABWs to address indicators of an SOC further.

In summary, there is a lack of research on the transition to ABWs that integrates perspectives from both managers and employees, concentrating on the SOC. Studying both groups is important as it may lead to a more comprehensive understanding of how people from different levels within the workplace may handle and adapt to the changes. Furthermore, it may reveal factors that can facilitate or act as barriers to a sense of coherence for both groups during an organisational change. Hence, the aim of this study is to explore how managers and employees experienced the transition period to an ABW through the lens of the SOC. Three research questions will be addressed:

1. How were comprehensibility, manageability and meaningfulness perceived by managers and employees in public sector workplaces during the transition to an ABW?
2. What are the identified barriers and facilitators during the transition to ABWs for managers and employees in public sectors seen from an SOC perspective?
3. Which practical and theoretical implications can be suggested based on the study findings?

## Research setting

During the autumn of 2023, the majority of the managers and employees in a Swedish mid-sized municipality moved into a newly built office building with an activity-based workplace design. In total, 620 administrative workers (534 employees and 86 managers) moved into the new building. They represented different departments: social care, the administrative office, the city planning office and culture and leisure. The purpose of the move was to set up the municipality’s activity-based way of working, continue to gain support for this approach amongst employees, and develop a foundation to prepare the functionality of the building for an activity-based working method. One of the aims was to lay the groundwork for creating an attractive workplace with an effective way of working, supported by the building’s features and IT solutions. After the relocation, the workforce was spread over three floors containing open-plan offices, three zones (creative, middle and silent zones), conference rooms, a canteen and coffee rooms. The relocation process started in 2019 with a project including workshops with employees and managers, field trips by managers to other organisations that had implemented ABWs and training by working in open-plan offices.

## Methods

### Study design

The research was designed as a qualitative content analysis. This consisted of individual interviews with seven managers in the municipality during the transition to the ABW, and two focus group interviews with nine employees from different administrative departments who recently started working in the ABW. The managers included both middle managers and first-line managers. Although their positions were at different hierarchical levels, both groups shared responsibilities for managing their workgroups, providing support and resources to employees to perform their tasks, and addressing occupational and health issues.

A qualitative design was deemed appropriate as the aim was to gain an understanding of how the participants made sense of the transition. Deductive reasoning, as described by Elo and Kyngäs [37], was applied during the analysis. This type of analysis can be used to help identify key concepts within the data and is used when there is previous knowledge about a phenomena and existing theoretical frameworks [37]. The SOC concept by Antonovsky [25] acted as the theoretical framework for the analysis process. The Consolidated Criteria for Reporting Qualitative Research (COREQ] checklist was used to enhance the reporting and methodological quality of the study [38].

### Participants

For the individual interviews, seven middle managers were purposively recruited by an assigned contact person in the organisation’s HR department. The inclusion criteria were that the participants were unit managers within municipal departments who had been part of the transformation to the ABW and had worked as managers before, during and after the transition. The contact person from the HR department shared an email with a list of 15 managers who fulfilled study requirements. All 15 managers were contacted via email together with a link to a booking page (Doodle). Seven of these chose to participate by booking a suitable time. Of the seven participants, six were women and their age varied from 43 to 55 years. They had been working in the organisation for between 2 and 23 years and as managers for between 2 and 9 years and led an average of 15 employees.

The employees were also recruited through an assigned contact person in the organisation’s HR department, who provided the email addresses of nine employees that met the study requirements and were available to participate in the focus group interviews. The inclusion criteria were that the participants were municipal employees who had been part of the transformation to the ABW and had worked in the traditional office design for a minimum of one year before the transition. Of the nine employees, eight were women. They worked in different departments (human resources, municipality office, cultural department, business development office, community development administration). No questions were asked about their age or the length of time they had worked in the ABW during the interviews.

### Data collection

Data was collected through semi-structured individual interviews and focus group interviews between February (two months after the relocation) and April 2024. Individual and focus group interviews were used in conjunction to yield in-depth data within and between groups [39]. From this perspective, focus group interaction might be viewed as more than a neutral conveyor of information, but as giving a deeper understanding of the phenomena (i.e., reflection on ABWs) [40]. According to Morse [41,42], multiple qualitative methods within a single study may enhance the analysis and create a deeper understanding of the phenomena.

The interviews with the managers were conducted by two female research assistants (BSc in Psychology) who introduced participants to the study and their reasons for conducting the study. The assistants had previous experience conducting qualitative research and received extensive input from professionals specifically related to the research methods and interview technique. The interviews followed a semi-structured interview guide covering topics such as background information, the transition, reflections about the process and leadership behaviours. A pilot interview was performed to test the relevance of the questions. Only a few adjustments were necessary, such as changing the order and reformulations of a few questions.

Two research team members conducted the focus group interviews: one male and one female, both holding PhDs and working as professors. The research team developed the interview guide, and all team members provided feedback to refine the clarity, relevance, and coherence of the questions. The interview guide covered topics such as preparations before the move, the implementation process, their daily work, leadership behaviours and the work environment. The interviews were audio-recorded and transcribed clean verbatim [43]. The individual interviews lasted an average of 45 minutes, and the focus group interviews lasted an average of 90 minutes. Neither of the interviewers conducting the individual and focus group interviews had a pre-existing relationship with the participants and non-leading, open-ended questions were used to mitigate potential bias during the interviews and to allow participants to express their perspectives freely.

### Data analysis

All transcripts were coded by the first author, after which the other authors thoroughly reviewed the transcripts and codes and provided detailed feedback. Only minor changes were made to the codes based on the review. The analysis was performed deductively in several stages, in accordance with Elo and Kyngäs [37]. The interview transcripts were initially read several times to make sense of the whole. A theory-based categorisation matrix with the pre-set categories of comprehensibility, meaningfulness and manageability was then created using the NVivo software (1.4.1). Once the categorisation had been created, all the data was reviewed for content, and condensed meaning units were coded deductively in line with the categories identified. The data from the individual interviews and focus groups were coded together and differentiated by identifiers to separate insights derived from the different groups.

Meaning units from both data sets were coded into the comprehensibility category when the participants discussed if and how they made sense of information or situations regarding ABWs. Meaning units were coded into the manageability category when the participants discussed the degree to which they felt that there were resources at their disposal and how challenges were handled. When statements concerned their motivation and desire to cope with the stimuli encountered, and their experience of meaningfulness in their work, the meaning units were coded into the meaningfulness category.

The coded meaning units in each category were then grouped and categorised into sub-categories according to their meanings and similarities. The most used sub-categories were chosen for further analysis: information and preparation, clear rules, adaptability, leadership, social relations, and health and well-being (Table 1) and these were divided into facilitating factors and barriers to obtaining a SOC. Facilitators were conceptualised as factors that enhanced the components of comprehensibility, manageability, or meaningfulness, and barriers were understood as factors that hindered these components, creating challenges in adapting to ABW.

**Table 1:** Coding framework based on the sense of coherence categories.

Category	Sub-category	Code	Managers Facilitators	Managers Barriers	Employees Facilitators	Employees Barriers
**Comprehensibility**	*Information and preparation*	Understanding before the move	“Then we worked in the group to absorb the information so that we would be prepared when we got here.”	“But it was still impossible to understand how it would turn out.”	“There were people in charge that you could ask during the “employee day”. It was very, very good. The information there, they did great.”	“We had to ask a lot of questions. You didn’t really understand what it would look like.”
	*Clear rules*	Unambiguous rules	“Then, as a manager, you have to stand up for the fact that no, this is how it is now.”	“Because we have seen that, and it is of course individual managers who have not hammered them (the rules) home into the working groups.”	I’m glad that this “has been taken up by the managers, because it is needed. What is okay in the middle zone?”	“Some people use the focus rooms as their own office anyway, you park yourself there. It’s one of the big obstacles.”
**Manageability**	*Adaptability*	Flexibility and adaptation	“One good thing is the variety of environments. Because working life is so flexible today. That you can adapt the situation to the place you are working in.“	“It takes more time (to move). I have to say that.”	“I like the zones. But that’s because I vary (in tasks).”	“Problematic if I had to be here all the time. At home, you can take sensitive issues, can sit and talk. But if everyone is going to do it on-site, then it needs to be expanded. “
	*Leadership*	Available leaders	“I am clear with information. I think it’s important. I am clear about preparations.”	“The biggest fear is probably that I feel a loss of control. I would like to have a very good overview of what my employees are doing.”	“For us, it has become that way (that the manager is a key person in the new way of working), we have a good manager.”	“How does the manager keep track? Great responsibility? Important responsibility! And how do they do it?”
**Meaningfulness**	*Social relations*	Staying in touch with each other	“We have one of those Teams channels where you can write, now I’ll go and have lunch, if anyone else is going to have lunch. Or now we’re having coffee, or are we going to... So we’re trying to do it, now we’re going to have an after-work thing tomorrow (...). We usually try to do things together.”	“It can be days before you meet someone. And a fear that some may fall under the radar.”	“We always write in teams when we go for coffee.”	“When you had your offices next to each other, you had control, but now you don’t have control – you may have only been working from home for two weeks. You can lie dead at home, and no one notices (...) And that is worrying.”
	*Health and well-being*	Work environment experiences	“Everyday exercise has definitely increased. There is a lot of walking.”	“You are careless about where you sit. How you sit. And what aids you use. It means you start to get a little pain here and there.”	“The week after (the moving-in party) there were happy faces in the corridors. Partying itself is about bonding.”	“You can end up with a chair that cannot be adjusted.”

### Ethics

Ethical approval of the study was given by Sweden’s Ethical Authority Board (Dnr: 2023-06718-1). Before each interview began, information about the study and the conditions for participation were given orally, and in writing. The researchers also ensured that all participants received, read, and understood the information before the interview started. All participants provided written and oral consent before participating in the study and were informed about their right to withdraw from the study without giving any reason. The data was stored properly according to the Swedish Act on Ethical Review of Research Involving Humans (SFS 2003:460).

## Findings

**Fig 1:**
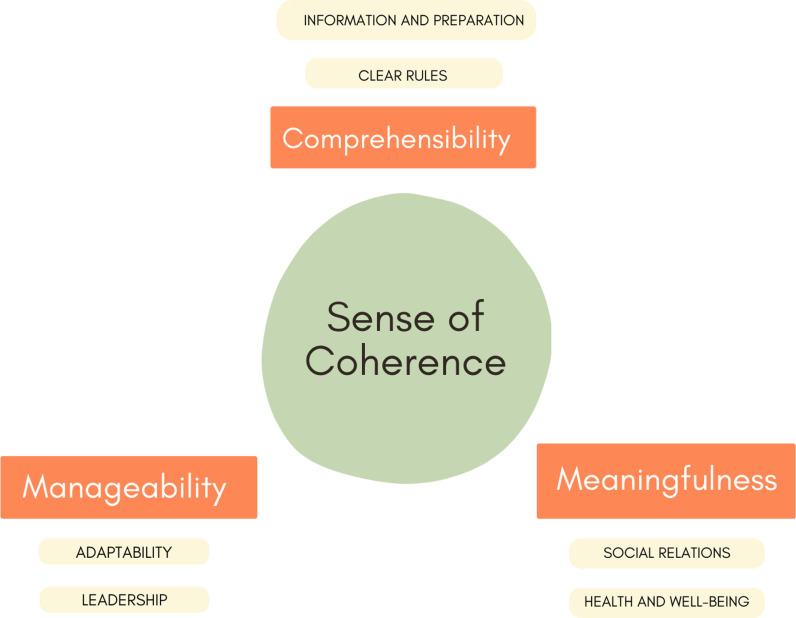
The three SOC categories and the sub-categories obtained from the analysis.

Six sub-categories within the three main SOC categories were identified in the material and considered important to the participants during the transition to ABWs (Fig 1).

### Comprehensibility

The first category relates to the participants’ level of comprehensibility, i.e., that incidents and circumstances are structured and understandable [25]. The category is built on the sub-categories of *information and preparation* and *clear rules* which were formed during the analysis. These represent how the participants received and made sense of information during the transition to the ABW.

#### Information and preparation.

##### Facilitating factors:

The analysis showed that the managers had been preparing for the move to ABWs for several years and that they had been given good information and material from their superior management levels. They also had assistance from a project manager who explained in detail how they should proceed with the move and what they could expect on-site. While they had to prepare for the move themselves, they also had to prepare their employees for the change. They provided meetings, workshops, information on the intranet, and staff development days to make the move more comprehensible for themselves and the employees.

One of the managers expressed this as follows: *“We have carried out a variety of exercises from sitting with health and safety representatives and filling out a risk and impact analysis in our control and management system, to having group discussions, and looking at the material that communicators and others in the building group have produced.”* Manager 3.

The analysis showed that the employees found the information and preparations before the move to be important for them to get insights into what the new work environment would look like. One thing that was seen as especially valuable was the “staff development days” where they were able to get more information about the move. They had an exhibition where they could be more “hands-on” and were able to touch and look at chairs and bags. They also had a lecture from a university researcher who shared some of the latest research about ABWs*.* The following excerpt is an example of one of the employees’ views about the lecture: *“She brought up many different aspects. She was very good. I had preconceived notions that they had requested a halleluljah lecture.”* IP 2, Group 1.

##### Barriers:

The analysis showed that both the managers and employees found it hard to visualize how the ABWs would work in practice and that many people were concerned about the outcome of the move, despite the information and workshops. What was evident in the material obtained from both groups was that a field trip would have increased the comprehensibility of the situation as some people may need more practical insight into the work environment. This was expressed by a manager:

*“One thing that would have been beneficial, and that we talked about doing but it never happened, was an employee field trip to the town hall. That would have been good.”* Manager 6.

One thing that was clear in the material was that the management communicated that the move to an ABW was done to improve collaboration and to have flexible premises, but that they failed to mention the financial aspect. The materials showed that transparency about why the change was needed, and communicating it clearly, had been desired. One of the employees stated:

*“It was never communicated that it was a financial issue.”* IP 3, Group 2.

#### Clear rules.

##### Facilitators:

In terms of ABWs that have clearly divided zones, the analysis showed that it was important for both managers and employees to understand the rules that apply in the different zones. The importance of going through the rules repeatedly to make them stick was a regular point raised in the material. This was expressed by one of the managers as follows:

*“It takes some time. But you need to do a tour and explain and demonstrate all the rules. And then it’s learning by doing. That you have to do it as you go along.”* Manager 6.

The material revealed that it was important for the employees to know what was expected of them and how to act in different zones and situations. Furthermore, it emerged that the employees had noticed that there were informal rules on the different floors and in different zones, and that this was somehow accepted in certain groups. For instance, this could involve rules about what you could do in the zones or where you could sit. The employees expressed that it was important to talk to each other to avoid these informal rules. One employee stated:

*“It’s very easy to mark territory and you can feel that no, can I sit here even though this is where they always sit? It’s very important to talk to each other.”* IP 3, Group 2.

In addition, it was expressed that having a manager who addressed issues, and who took misconduct seriously, increased the understanding about what was acceptable to do in the workplace and what was important for the work environment.

##### Barriers:

One finding that emerged from the analysis was that certain managers did not reinforce the rules effectively, which may have been due to a lack of clarity about the rules. One manager discussed the need for managers to emphasize the significance of rules in the working group: *“Because we have seen that individual managers have not properly hammered them (the rules) home into the working groups.”* Manager 1.

The analysis revealed that some people did not follow the rules and, for instance, used the quiet focus areas as their own offices, or spoke loudly with colleagues when other people were working. This irritated many people and had a negative effect on their ability to focus. Some also said that what was permitted in the different areas was still unclear: *“There are some ambiguities about the zones. Can you have a hybrid meeting in the creative zone?”* IP 2, Group 1.

### Manageability

The second category, manageability, identifies the extent to which the participants perceived that they had the requisite resources to cope successfully with the challenges, and the extent to which they invested time and energy in finding solutions to these [25]. The category is built on the sub-categories *adaptability* and *leadership*, which emerged from the analysis. These show how the managers and employees adapted to different situations and how leadership was experienced by employees during the move to the ABW.

#### Adaptability.

##### Facilitators:

A central finding in the material was that the flexibility of ABWs was deemed to be good for productivity for some managers. The fact that it was possible to choose workspaces depending on the work tasks was generally seen as positive. One manager expressed that the different zones increased their ability to categorize and structure work: “*You get better at categorizing your work... You have to sort of structure your way of working. I think that’s positive.”* Manager 2.

Furthermore, the analysis revealed that the different zones were beneficial when used properly. It was also found that the flexibility of working from home when needed increased the employees’ ability to handle work and different tasks, as well as their work-life balance. One employee expressed that being able to telework was important for many people: *“I know several colleagues who would change jobs if they had to be at work every day.”* IP 4, Group 2.

##### Barriers:

The analysis revealed that for both managers and employees, one of the negative aspects of ABWs was that it could get very crowded on some days, and many people occupied the spaces. This could lead to people not being able to sit in the zone or spot most suitable for their work task. The following excerpt is a quote from an employee describing this: “*It’s very busy at times. More crowded than expected*”. IP 3, Group 2.

In addition, employees expressed that it could be quite time-consuming and inconvenient to pack up and change places several times during the workday.

Another issue that was revealed during the analysis was related to privacy. The employees stated that there was a lack of confidentiality in the middle zone due to the arrangement of the workstations, with computers angled so that screens could be seen by others. Furthermore, the privacy filters on the computer screens only worked when viewed from the side but not from directly behind, making it stressful when managing matters of confidentiality. One employee said:

*“It’s a bit stressful when someone walks behind me (…). I can’t sit just anywhere with confidential matters”.* IP 2, Group 2.

#### Leadership.

##### Facilitators:

It was evident from the material that most managers trusted their employees and believed that they had the knowledge and competence to perform different tasks. The material also showed that they delegated many tasks, which was essential for them to be able to manage their own workload. Even though the employees had a lot of freedom and were independent, they also appreciated having a close dialogue and making sure that they could get the support they needed to manage different tasks.

*“Working together is more like what I think. And that it is about freedom that comes with a responsibility”.* Manager 1.

The employees found the managers to be key persons in the ABWs and that it was important to have a very good leader who works with their employees and is clear with expectations and responsibilities. When the managers helped employees adapt to changes in the new work environment, they felt that they were able to manage their work tasks more effectively. They also stated that it was important for managers to act and deal with colleagues who do not behave appropriately, as otherwise it could affect their ability to manage their work.

*“It is up to each manager to handle employees that it doesn’t work out well with.”* IP 2, Group 2.

##### Barriers:

The analysis showed that the managers experienced a lack of control over the employees compared to cellular offices. They found it easier when members of staff worked in the same place all the time as it was simpler to identify problems and keep track of how the employees were doing. One manager stated:

*“From a selfish leadership perspective, it would be easier to have the people around me.”* Manager 4.

Through the analysis it became evident that the employees also experienced that their managers had less control over the employees in ABWs, where they work from home or often change workstations. Some stated that they regularly had to go to the manager themselves with issues and that the manager did not come to them. Just like the managers, the employees highlighted that it is harder for the leaders to monitor the employees in ABWs. One employee expressed this in the following terms:

*“How do you know how people are feeling? Before, you met every day. How does the manager keep track? It’s a great and important responsibility! And how do they do it?”.* IP 4, Group 2.

### Meaningfulness

The third category identifies whether the participants were able to find meaning in the situation, i.e., the extent to which an individual believes that life makes sense emotionally, and that they possess the motivation and desire to cope with any stimuli encountered [25]. This category is built on the sub-categories *social relations* and *health and well-being*, representing how they made sense and meaning of their situation during the move.

#### Social relations.

##### Facilitators:

The analysis showed that the managers and employees believed that the increased mobility and higher number of common areas meant that they could more easily meet other individuals and have meaningful and rewarding conversations with them. It was revealed that several of the interviewees had started talking to new people in different areas of the building and made new connections. One manager stated: *“So the main benefit is that you can come across someone and it leads to good outcomes. By ending up at the same desk, or part of some area, you start talking about something. Or pick up something. And it can have a very good result. Or just that it’s pleasant for the work environment.”* Manager 2.

In addition, both groups highlighted the importance of keeping in touch with each other regularly as they are less likely to see each other in ABWs compared to cellular offices. Having chat groups where they indicated who was at work and when they are taking a coffee break or lunch was seen as important for the group’s cohesion and work environment. One employee mentioned this in the following way:

*“We have a chat group for our department named “Who is here?””* IP 2, Group 2.

##### Barriers:

Throughout the material, many managers expressed that the lack of cellular offices decreased their ability to “pop in” and have spontaneous conversations with colleagues, and that it affected their ability to build strong relationships with them. In addition, not knowing where and what the employees were working on decreased their sense of control. One manager expressed their thoughts as follows: “*And it’s still quite a large working group to feel that you have control over. Some are great at getting in touch and some are not. And you might not see someone (in person) for several weeks, even though you see that they’re here. It’s a challenge.”* Manager 1.

Similarly, the analysis showed that the employees felt there was a risk of some people falling off the radar in ABWs. For instance, it was hard to know whether someone was working from home or whether something had happened to them. In addition, employees mentioned that some people might feel lonely and excluded in an ABW if they are not invited to coffee breaks, lunches, etc. This was expressed by one employee as follows:

*“It can get very lonely if you are not invited via Teams”* IP 2, Group 1.

#### Health and well-being.

##### Facilitators:

The analysis revealed that the managers and employees believed that they were more physically active during the day in an ABW than in a cellular office, and several had noticed that they took more steps since moving into the ABW. The fact that they had to move around more frequently prevented them from remaining seated, which they believed had a positive impact on their health and well-being. One manager said:

*“I read a study recently that said research has found that it is positive (for your health) if you have a workplace where you need to move a lot if you otherwise have a sedentary job. And I think that you really do that here.”* Manager 2.

A further aspect that had a good impact on many people’s well-being and quality of life was being able to choose whether to work from home or from the office. While being at the office, meeting colleagues and moving around gave many people energy, the possibility to work from home occasionally was also seen as important for mental recovery and the work-life balance. One employee with a long commute to work said:

*“But what I feel is that it’s a luxury being able to be at home, (...) that is my big win. I get a much better personal life because I don’t have to go in to work every day. I enjoy working here. I get a better quality of life, much better.”* IP 4, Group 2.

The material showed that having a large welcome party to which all staff in the ABW were invited had a good impact on the employees’ well-being. The employees believed that the party strengthened the feeling of cohesion of the group and that they hoped that this would be a recurring event. One of the employees said:

*“I’ve never had so much fun”.* IP 4, Group 2.

##### Barriers:

One prominent aspect of the material was that both managers and employees found that having to change seating areas so often and sitting in places that were not adjusted to them, was a major problem of the move. They also had to carry around backpacks and heavy equipment during the day when they changed seats. To overcome this, many people often used their laptops, without a keyboard and a mouse, which caused physical discomfort due to prolonged use in unergonomic positions. One manager expressed this as follows: “*The disadvantage, a big disadvantage of moving here, is that I often just walk around with the laptop and sit with it. It’s a huge problem. I have to wear a wrist support, and I guess that’s because of this.”* Manager 1.

The majority of the managers and employees also reported that it was hard to find chairs that were suitable for them, and that it was hard to adjust them. Sitting on poorly adapted chairs and at tables that were not well-adjusted caused physical problems such as back and neck pain. One employee described that she had a hard time adjusting the chairs due to lack of strength:

*“It’s like it’s really complicated to set up the chairs (...). The chairs are really hard to adjust and you’re lucky if you find one that suits your body. But to adjust them... It takes a really long time and it’s really difficult. I never get it right because I’m too weak.”* IP 3, Group 1.

## Discussion

The aim of this study was to explore how managers and employees experienced the transition period to an ABW through the lens of an SOC. This is an important research focus, as studies of ABWs often lack a health-promoting perspective.

The main finding of this study is that managers and employees reported both positive and negative experiences during the transition to an ABW, influenced by factors that acted as both facilitators and barriers to an SOC. Although both groups identified several similar facilitators and barriers, differences emerged due to their hierarchical positions. Managers generally had more opportunities to act and influence workplace conditions, directly affecting employees’ experiences of the work environment. Employees, on the other hand, had less power to influence these conditions but adapted to challenges through individual strategies.

The strength of an individual’s SOC is shaped by both negative and positive life events, as well as internal and external resources [25,44]. These findings suggest that to strengthen SOC among managers and employees during the transition to ABWs, it is important to enhance positive factors and resources while addressing the negative aspects of the work environment. Similar conclusions have been drawn in previous research on the implementation of ABWs [36]. Even if a person is exposed to stressors or new situations, a sufficient level of comprehensibility, manageability and meaningfulness may enable people to feel self-efficacious to deal with the situation and to identify resources in the environment [25,44,45].

Based on the results presented, it can be concluded that it is important for managers and employees to be provided with both theoretical and practical information to better comprehend and accept the situation when transitioning to an ABW. For the managers, receiving structured information from superior management enabled them to pass on information to the employees. Workshops, seminars and lectures facilitated the information sharing and made the processes clearer leading up to the move, which was very important for both groups. This is in line with a study by Bergsten et al. [1] which concluded that information and support is needed when relocating to ABWs to achieve employee satisfaction with the relocation. The study also found that the employees gained an increased understanding of the ABW concept when they took part in activities such as workshops and seminars surrounding the relocation.

In accordance with other studies, involvement and support from managers are important for the employees’ perception of a new intervention [1] and starting the preparations early facilitates mental preparation and acceptance of change [46]. The results reveal that when transitioning to ABWs, communication about why the change is needed is essential. Failing in this communication might decrease the comprehensibility of the situation, as emphasized by the employees in our study. Previous research has shown that clear goals for the change are important during organizational change processes related to workplaces [47,48]. Open discussion between managers and employees is needed as the latter group may resist change if the goals are not understood [48].

In terms of ABWs with clearly divided zones, the results confirmed that both managers and employees need to understand what applies in the different zones, and the managers have an important role in communicating the rules. One study found that when the rules in ABWs were explicit and clear, it resulted in a higher proportion of resources in terms of positive work conditions [49]. Furthermore, clear rules made people feel secure in their actions, while the absence of clear rules led to different interpretations of acceptable actions and rule-breaking. Another study suggested ongoing supervision on the proper use of the work environment for ensuring employees use the premises as expected [50], which was also confirmed in the interviews with the participants in our study.

Our analysis indicates that factors such as flexibility and leadership affected the managers’ and employees’ feeling of manageability. While changing workstations often in the ABW can be time-consuming, the flexible work and different zones may be beneficial as they can help people structure their work more effectively, increase autonomy, and enhance people’s work-life balance. Mache and colleagues [11] also reported that job autonomy increased after the transition to ABWs. Their analysis revealed that ABWs may be challenging for managers as it can reduce the ability to monitor, check and coordinate with their employees. As ABWs may increase freedom and autonomy for workers [11] and decrease contact between managers and employees [12], it may be important to maintain a close dialogue to make sure that there is clarity about what is needed from both parties to manage different tasks [11]. Research has pointed toward the importance of a leadership style that promotes trust between managers and employees in ABWs [12], which was also emphasised in the interviews in our study. If managers show only limited trust and try to limit the autonomy of employees, it may negatively affect employee well-being, job satisfaction and job performance [51]. Finding ways to check in on each other in a flexible work environment is important. According to Harris [52], effective communication between managers and employees is important for teamwork, the delegation process, job structure and planning. For this reason, in ABWs communication needs to be done using different channels to increase its effectiveness [53].

Social relations and health and well-being may affect the level of meaningfulness managers and employees find in an ABW. The interviews revealed that the ABW made it easier to network and have meaningful conversations with different people in the office building, but also harder to keep in touch with people in the immediate team. This is consistent with other studies that found that ABWs negatively impacted social interactions within the teams [54] and led to a decline in satisfaction with communication, and a decline in the sense of belonging post-relocation [13]. Conversely, the results are in contrast with the findings from a study by Häne and colleagues [55] where the ABW was reported to improve staff interaction, communication and collaboration. As people moved towards working more from home and in different places during the day, it seemed inevitable that fewer social interactions would occur. Finding ways to stay in touch with colleagues regularly was important to the cohesiveness of the team in our study. Given that humans derive meaning from their social relations, which is critical to their health and well-being [56,57], this is an important issue to address. Participating in parties and activities both outside and inside the immediate work circle was demonstrated to strengthen cohesion in the workplace. Social well-being in the workplace includes engaging in enjoyable activities, which can enhance meaningfulness for workers [58,59]. This may help to improve performance, form a positive job attitude and bring about proactive behaviours [60,61].

According to our analysis, the ABW led to increased physical activity, which may have a positive effect on the workers’ health and well-being, which has also been reported in other studies [7,62]. Conversely, ergonomic issues such as sitting poorly and carrying heavy equipment were reported by managers and employees alike, which is in line with other studies [2]. This in turn may lead to health issues and distract people from work [63]. The physical work environment is important for the workers’ health, well-being and job satisfaction, and is subsequently connected to meaningfulness [64]. To increase meaningfulness the management should work together with the managers and employees to create meaningful office environments where people feel valued and supported [65].

Moreover, in addition to identifying facilitators and barriers in the implementation of ABWs, the analysis can be indirectly linked to how favourable working conditions enhance productivity outcomes. A productive and sustainable work environment requires adequate access to information, clearly defined rules, flexibility, available managers, health-promoting working conditions, social activities, and a structured approach to social relations [1,66–68]. In addition, if employees experience the transition processes as satisfactory and meaningful, it increases self-rated productivity [69].

The knowledge obtained from this study may support organisations in the implementation of ABWs. Considering the identified facilitators and barriers could enhance the transition process.

## Conclusions and implications

The transition to ABWs from traditional offices is a complex process and the managers and employees had both positive and negative experiences during this period. The analysis showed that they were exposed to factors which may have acted as both facilitators and barriers to an SOC. According to the analysis, the participants’ comprehensibility during the transition was affected by information, preparations and clear rules. Flexibility at the workplace and leadership appear to have affected the managers’ and employees’ feeling of manageability. Finally, the analysis indicated that social relations, health and well-being affected the managers’ and employees’ perceptions of meaningfulness.

To strengthen the SOC in managers and employees during the transition to ABWs, there needs to be a focus on increasing positive factors and resources as well as addressing the negative factors in the work environment. A sufficient level of comprehensibility, manageability and meaningfulness may enable people to feel self-efficacious enough to deal with the situation and to identify resources in the environment [44].

It is challenging to propose universally applicable strategies for transitioning to ABWs with an increased proportion of employees and managers working from home. Workplace-related ABW interventions must consider the specific context and nature of the work when implementing such strategies [70]. Nevertheless, certain recommendations can be more broadly applicable. Based on the deductive analysis using the SOC framework, the results suggest that adequate information and preparation both before and during the transition to ABWs may facilitate employees’ and managers’ experience of the process as more meaningful and comprehensible. Additionally, it is essential to establish clear guidelines for both employees and managers concerning the use of various work zones. Conducting workshops and seminars that provide practical guidance on the effective design of ABWs, address ergonomic concerns, and support the maintenance of relationships between employees and managers can enhance the sense of meaningfulness for all stakeholders involved.

A critical factor for both meaningfulness and comprehensibility is the implementation of a planning phase before the transition, which includes several mandatory activities for both employees and managers. If participation in these activities is not compulsory, there is a risk that non-participating individuals may perceive a lack of information about the transition as a significant barrier. In terms of manageability, it is essential for employees to have adequate resources to navigate the challenges, such as access to different work zones and the flexibility to alternate between working from home and the office. To promote high levels of employee meaningfulness, comprehensibility, and manageability, it is essential for managers to engage in supportive and adaptive behaviours during and after the implementation of ABWs. Furthermore, providing managers with sufficient knowledge and resources is crucial for exercising effective leadership in the context of ABWs. Lastly, to support manageability for both groups, it is important to design ABWs that uphold high standards in physical work environment factors, including well-designed workspaces, ergonomic equipment, and effective noise reduction measures.

For future research, both qualitative and quantitative studies with larger samples are required to gain a better understanding of the effects of the SOC on employees and managers when transitioning to ABWs. Studying other organisations to enhance the understanding of the SOC in different settings is important. Further research is needed to explore how the intensity and quality of preparation phases are associated with SOC outcomes for employees and managers. Additionally, future studies should examine how various types of work and individual factors, such as gender, age, and family circumstances, influence attitudes toward ABWs and how they affect health and productivity outcomes.

## Strengths and limitations

Previous studies on the SOC in the workplace have mostly focused on traditional offices, whereas this study explores the SOC in the context of ABWs and provides insights into how the SOC can be affected in flexible work environments. Moreover, a strength of the study is that it included both managers and employees, which to our knowledge has not previously been done in relation to the SOC and ABWs. The extensive interviews made it possible to deepen the understanding of the participants’ experiences during the transition to ABWs. Using both individual interviews and focus group interviews acted as method triangulation and provided insights of the area from both an individual and group perspective. Further, to enhance trustworthiness, Lincoln and Guba’s approach of credibility, transferability, dependability, confirmability and authenticity was used in this study [71]. Credibility was achieved through data-source triangulation from the individual and focus group interviews. Transferability was achieved by giving a clear description of methods and participant characteristics. Dependability was achieved through transcript review and discussions in the research team. To ensure dependability and confirmability, reflective thoughts after interviews, notes on decisions taken during the research process, and research materials were saved to allow for a review of the transparency of the research path. Finally, authenticity was demonstrated through the verbatim transcriptions and inclusion of participant quotes. Reflexivity was continuous throughout the research process and encompassed the authors reflecting on their impact on the analysis and their personal biases.

Limitations of the study may be that the results reflect local conditions in Sweden that may not be representative of other cultural contexts in Europe. However, the results may be transferrable to similar Swedish public sector workplaces. Furthermore, the participants came from one organisation making it hard to generalise the findings to other workplaces. However, they represented different and common departments in municipal organisations. The participants were purposively selected, and it is possible that participants who were more positive about the transition chose to participate. The pre-defined SOC categories may have implied a potential disregard for other relevant categories. However, the categories were not used to guide data collection or probe questions during the interviews and were only applied during the analytical process. The coding process remained data-driven within the framework. The small sample size of seven managers and nine employees may also be considered a limitation. However, the material was rich [72], hence adequate [73], and was therefore deemed sufficient to answer our research questions.

Lastly, the use of both individual semi-structured interviews and focus group interviews may be seen as a limitation as individual semi-structured interviews generally offer in-depth conversations, whereas focus group interviews offer multiple perspectives at once and might be less in depth. Nevertheless, different qualitative methods within the study were used as it may enhance the analysis of a phenomenon and broaden its conceptualisation [42].
